# First Ancient Mitochondrial Human Genome from a Prepastoralist Southern African

**DOI:** 10.1093/gbe/evu202

**Published:** 2014-09-10

**Authors:** Alan G. Morris, Anja Heinze, Eva K.F. Chan, Andrew B. Smith, Vanessa M. Hayes

**Affiliations:** ^1^Department of Human Biology, University of Cape Town, South Africa; ^2^Department of Evolutionary Genetics, Max Planck Institute for Evolutionary Anthropology, Leipzig, Germany; ^3^Laboratory for Human Comparative and Prostate Cancer Genomics, Garvan Institute of Medical Research, Darlinghurst, New South Wales, Australia; ^4^Department of Archeology, University of Cape Town, South Africa; ^5^Genomeic Medicine Group, J. Craig Venter Institute, La Jolla, California; ^6^Central Clinical School, The University of Sydney, Camperdown, New South Wales, Australia; ^7^Department of Urology, University of Pretoria, South Africa; ^8^Medical Faculty, University of New South Wales, Randwick, New South Wales, Australia

**Keywords:** ancient DNA, mitochondrial genome, Khoesan, southern Africa, marine foragers, archeological skeletons

## Abstract

The oldest contemporary human mitochondrial lineages arose in Africa. The earliest divergent extant maternal offshoot, namely haplogroup L0d, is represented by click-speaking forager peoples of southern Africa. Broadly defined as Khoesan, contemporary Khoesan are today largely restricted to the semidesert regions of Namibia and Botswana, whereas archeological, historical, and genetic evidence promotes a once broader southerly dispersal of click-speaking peoples including southward migrating pastoralists and indigenous marine-foragers. No genetic data have been recovered from the indigenous peoples that once sustained life along the southern coastal waters of Africa prepastoral arrival. In this study we generate a complete mitochondrial genome from a 2,330-year-old male skeleton, confirmed through osteological and archeological analysis as practicing a marine-based forager existence. The ancient mtDNA represents a new L0d2c lineage (L0d2c1c) that is today, unlike its Khoe-language based sister-clades (L0d2c1a and L0d2c1b) most closely related to contemporary indigenous San-speakers (specifically Ju). Providing the first genomic evidence that prepastoral Southern African marine foragers carried the earliest diverged maternal modern human lineages, this study emphasizes the significance of Southern African archeological remains in defining early modern human origins.

Southern Africa has arguably the richest and oldest fossil record of anatomically modern human existence outside of east Africa (Mitchell 2002; Brown et al. 2009, 2012; Marean 2010). The first genetic evidence for the significant role southern Africa has played in modern human evolution was provided using patterns of DNA variation in the maternally derived mitochondrial DNA (mtDNA) of contemporary populations (Chen et al. 1995; Ingman et al. 2000; Lombard et al. 2013). Concurring with archeological estimations (McDougall et al. 2005), mtDNA-derived molecular genetic age estimations place modern human emergence around 200 ka (Behar et al. 2008). Sequencing of complete mtDNAs from contemporary populations has dramatically improved the resolution of the global human maternal phylogenetic tree. The first emerging major haplogroup L0d is estimated to have split from the remaining L0-lineages around 150 ka (Behar et al. 2008; Soares et al. 2009). Today this earliest diverging extant maternal lineage is largely restricted to Southern African populations, in particular the click-speaking forager or Khoesan peoples (Gonder et al. 2007; Tishkoff et al. 2007; Behar et al. 2008; Barbieri et al. 2013; Schlebusch et al. 2013; Petersen et al. 2013).

Archeological evidence suggests herding migrants with sheep entered northern Namibia around 2,200 years ago (ya) migrating southwards along the western coast (Robbins et al. 2005; Pleurdeau et al. 2012), reaching the southeastern Cape by 2,000 ya (Sealy and Yates 1994; Henshilwood 1996; Smith 2006) (fig. 1). The living descendants from these early herding-forager migrants (the modern Khoekhoe) speak a Khoe–Kwadi language, which is different from the Ju-‡Hoan and Tuu languages spoken by the indigenous hunter-foragers (Haacke 2002; Güldemann 2008) and the nonclick-derived Bantu languages of the agro-pastoral migrants who arrived in the region roughly 500 years later through an eastern coastal route (Huffman 1992; Güldemann and Vossen 2000). Recent genetic analysis suggests a link between these earliest pastoralists and east Africa (Pickrell et al. 2012), with further ancient west Eurasian contribution to the Khoe–Kwadi speakers (Pickrell et al. 2014). Although skeletal remains demonstrating Khoesan morphology appear to be absent north of the Zambezi River (Morris 2002, 2003), extensive evidence exists for Khoesan inhabitance predating pastoralism at the most southern coastal regions. Successful extraction of DNA from the prepastoral archeological record has until now been hampered by extensive DNA degradation caused by high temperatures and acidic soil conditions (Smith et al. 2003). In this study, we report the first complete ancient mitochondrial genome from a prepastoral indigenous inhabitant from the most southern tip of Africa.

**Fig. 1.—:**
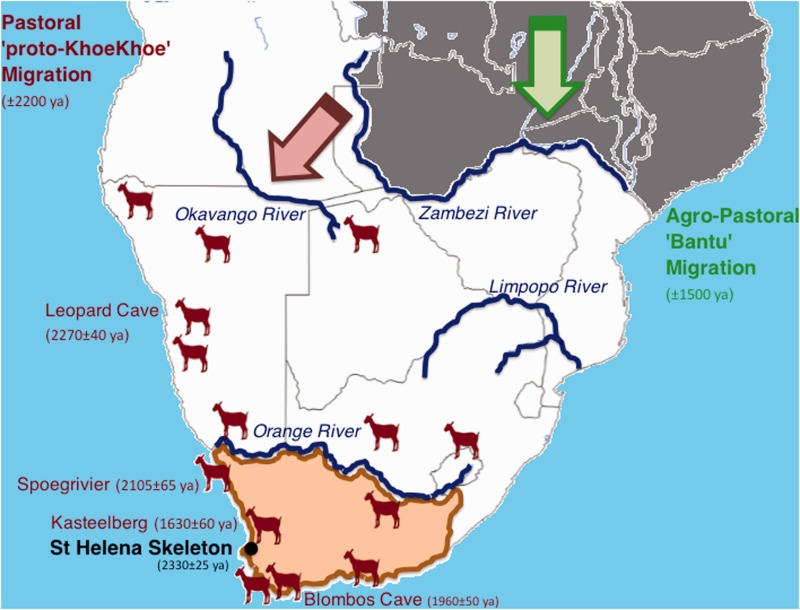
Map of southern Africa between 2,300 and 1,500 ya. Khoesan remains provide evidence for indigenous inhabitance across the entire region south of the Zambezi River (white), while absent north of the Zambezi River (gray). Prepastoral Khoesan remains have been found across the focus region of this study, that is south of the Orange River (beige), including the burial site for the St Helena marine forager skeleton (black). Sheep symbols indicate localities of sites with evidence of prehistoric pastoralism (adapted from Pleurdeau et al. 2012), with significant sites indicated (maroon). Archeological evidence therefore suggests that “proto-Khoekhoe” pastoralists migrated along a west coastal route southwards through Namibia before crossing the Orange River into South Africa. This migration was followed roughly 500 years later by the southward migration along the eastern coast of the agro-pastoral Bantu peoples (green).

In June 2010, an intact skeleton (UCT 606) was excavated along the southwest coastal region of South Africa at St. Helena Bay (32 °45′37″S: 18 °01′47″E; supplementary fig. S1, Supplementary Material online). The body had been placed on an impermeable consolidated dune surface, on its right side in a fully flexed position (fig. 2*A*). The bones originate from a single male who stood no more than 1.5 m in height. Dental wear and significant areas of osteoarthritis suggest that he was at least 50 years of age at time of death. Lack of any evidence of tooth decay and excessive occlusal wear suggests a diet typical of hunter-gatherer subsistence. The presence of abnormal bone growths in the right auditory meatus (ear canal opening) caused a condition known as “surfer’s ear” (auditory exostosis) and provides evidence that this individual most likely spent considerable time in the cold coastal waters sourcing food (Crowe et al. 2010). No obvious cause of death was evident. The results of carbon-14 to stable carbon-13 isotope ratio analysis of a rib provided an uncalibrated date of 2,330 ± 25 ya (sample ID: UGAMS 7255) with a δ^1^^3^C value of −14.6‰. The high concentration of shells found within the grave shaft provided further evidence for marine subsistence. The date calibrated to two standard deviations falls between 2,241 and 1,965 years before present (Dewar et al. 2012). This is calculated on data from the OxCal calibration programme corrected for an assumed 52.5% marine diet determined from the placement of −14.6‰ in the range of δ^1^^3^C values for western Cape skeletons (Dewar and Pfeiffer 2010). Although the minimum date falls right on the edge of the arrival of pastoralism in the Western Cape, anatomical and archeological analysis of this skeleton and the associated burial site clearly defines this individual as an indigenous Southern African, predating pastoral arrival into the region.

**Fig. 2.—:**
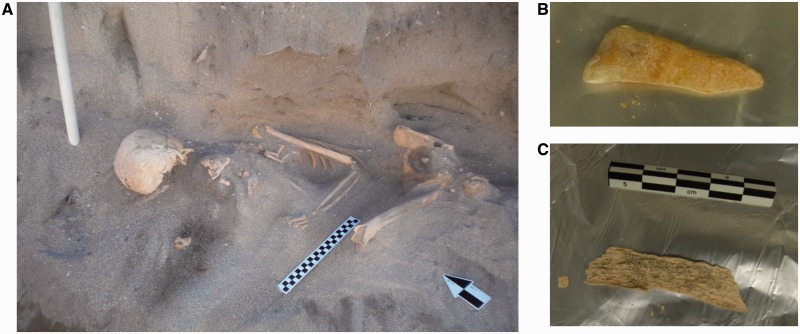
Burial site and skeletal remains of the St. Helena marine forager carbon dated to 2,330 ± 25 years before present. (*A*) The complete skeleton exposed during the June 2010 excavation revealed that this 1.5-m-tall male marine hunter was at least 50 years old at time of death. (*B*) The tooth and (*C*) single rib provided for ancient DNA extraction were not handled directly during removal from the burial site to minimize contamination.

DNA was successfully extracted from the largely protected inner canal region of a single tooth (fig. 2*B* and supplementary fig. S2, Supplementary Material online) and notably more degraded rib (fig. 2*C*). Mitochondrial genomes were sequenced using paired-end Illumina GAIIx sequencing and assembled from 34,274 (3.4% tooth) and 6,114 (1% rib) sequencing reads, yielding an average coverage of 103.1- and 20.8-fold, respectively (supplementary fig. S3, Supplementary Material online). No mtDNA position was covered by fewer than two sequencing reads. The consensus sequences of the tooth and rib were identical. To assess contamination rate with modern human mtDNA, we identified 32 “diagnostic positions,” where the same base was shown to be different from the ancient sample in at least 99% of a worldwide panel of 311 modern human mtDNAs (Krause, Briggs, et al. 2010; https://github.com/udo-stenzel/mapping-iterative-assembler, last accessed December 2013 and for www.phylotree.org, last accessed July 29, 2014). Four sequences from the tooth (of 1,678 covering the 32 diagnostic positions) and none from the rib (of 391 covering the diagnostic positions) matched present-day human mtDNA sequence at these positions resulting in a contamination estimate based on the upper endpoint of the 95% confidence interval approximated by the Wilson score interval of 0.6% and 1%, respectively. Further validation of integrity of ancient DNA was provided by the presence of nucleotide misincorporation reflecting cytosine deamination, a feature typical of ancient DNA (Pääbo et al. 2004; Briggs et al. 2007; Brotherton et al. 2007; Green et al. 2009), affecting more than 35% of cytosine residues at the ends of the DNA molecules (fig. 3). The average lengths of the DNA fragments, 50 bases for the tooth and 56 bases for the rib (ranges depicted in supplementary fig. S4, Supplementary Material online), are at the lower end of the range previously observed for much older remains of archaic humans (Briggs et al. 2009; Krause, Briggs, et al. 2010; Krause, Fu, et al. 2010).

**Fig. 3.—:**
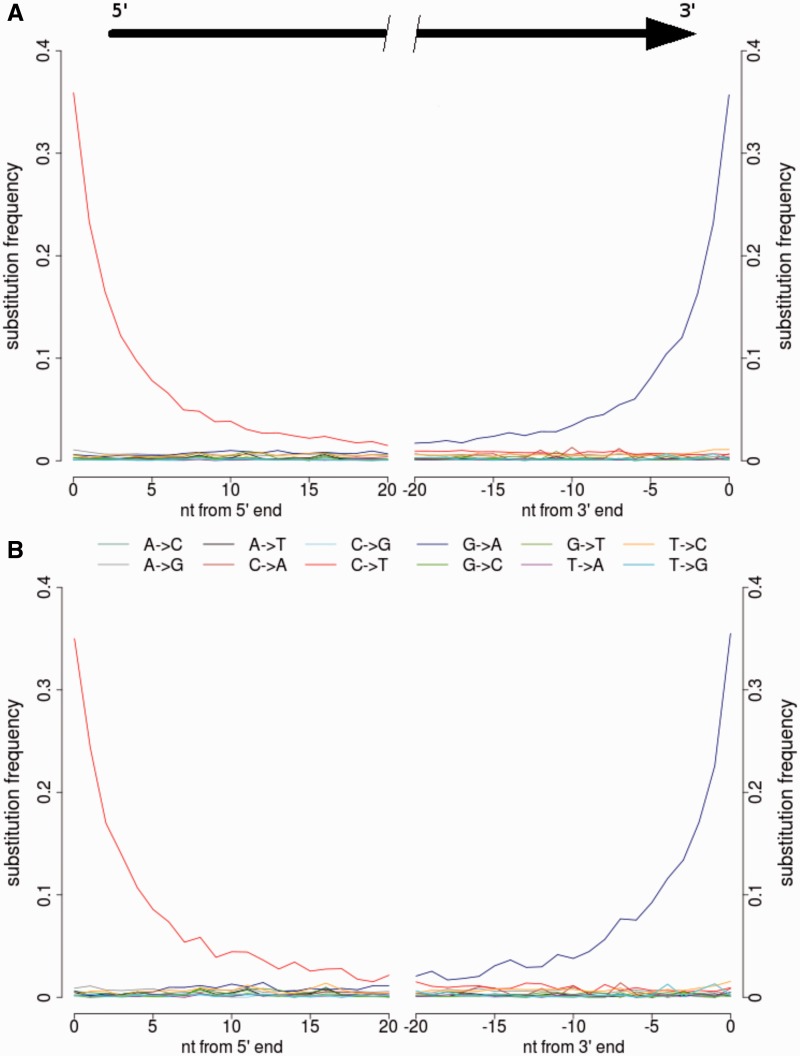
Substitution frequencies (patterns of DNA damage resembling ancient DNA) at fragment ends of mtDNA from the StHe used to assess present-day human contamination. The frequencies of the 12 possible mismatches are plotted as a function of distance from 5′- and 3′-ends of the sequencing reads. Substitution frequencies, X→Y, are calculated as the proportion of sequencing reads carrying the alternate allele (Y) to the human reference sequence (rCRS) allele (X). Deamination patterns of mtDNA sequence derived from the (*A*) rib and (*B*) tooth suggest the first successful extraction and sequencing of an ancient indigenous coastal Khoesan mitochondrial genome, while generating 100% concordant consensus sequences.

The complete ancient mtDNA was merged with 525 published complete genomes of regional and lineage relevance. These include 491 L0d/L0k donor-specific (Schuster et al. 2010; Barbieri et al. 2013), 26 L0a and seven L0f mtDNAs (GenBank), anchored to the revised Cambridge Reference Sequence (rCRS; van Oven and Kayser 2009). Phylogenetic inference as per PhyloTree Build 16 (www.phylotree.org, last accessed July 29, 2014) identified the St Helena skeleton (StHe) as belonging to the early-diverged L0d2c haplogroup, specifically L0d2c1 based on genomic similarity between the ancient mtDNA and the 12 publically available L0d2c1 genomes (fig. 4). The StHe mtDNA was most closely related (>99.9% similarity) to two Ju-speaking !Xun derived mtDNAs (NAM117 and NAM168), forming a new subclade L0d2c1c (defining variants C10822A and C16355T), that appears to have arisen independently from known subclades L0d2c1a and L0d2c1b. The order of L0d2c1 subclade emergence cannot confidently be determined given sample size, but overall results are similar when comparing whole mtDNA (fig. 4) and coding region restricted phylogenetic analysis (supplementary fig. S5, Supplementary Material online). Unlike the Ju-language predominance of L0d2c1c, both L0d2c1a and L0d2c1b lineages were represented by Khoe-speakers. Within this new lineage, nine unique variants (excluding the hotspot polyC 310 site) separate the ancient mtDNA from the two contemporary !Xun mtDNA including T408A, A2581G, A4824G, C11279T, C11431T, A11884G, T16086C, C16261T, and A16399C, whereas the contemporary genomes differ from each other at a single site, G3591A.

**Fig. 4.—:**
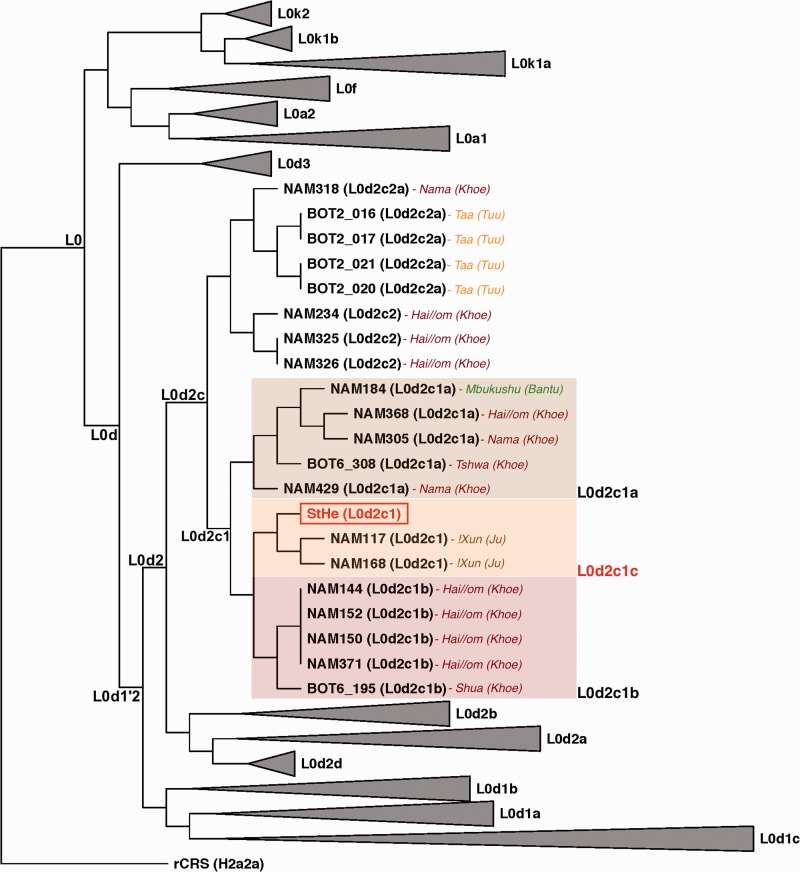
Phylogeny of 526 complete mitochondrial genomes depicting the earliest diverged modern human maternal lineages, including the first ancient Khoesan mtDNA (StHe) within the L0d2c lineage. All non-L0d2c genomes have been collapsed with each triangle representing the relative diversity of the corresponding haplogroups and subclades.

Although the precise birthplace or mechanism of Anatomically Modern Human (AMH) emergence is still debated (Weaver 2012), consensus has been reached that 1) modern humans originated within Africa, and 2) the most divergent (genetically distinct) contemporary human populations are found within southern Africa (Li et al. 2008; Henn et al. 2011). The first whole-genome sequence of a Khoesan individual shed light on the extent of this diversity (Schuster et al. 2010) and provided an early divergence estimate of 157–108 ka (Gronau et al. 2011). We generate in this study the first complete ancient Khoesan mtDNA, identifying not only a new early derived maternal lineage, but confirm through archeological and osteological analyses that ancient maternal human lineages were present in Southern African marine foragers prior to the arrival of southward migrating pastoralists. We conclude that further sequencing of the southern African archeological record is likely to expose additional unclassified human genome diversity.

## Materials and Methods

Permit for excavation of the skeleton was granted to A.B.S. under the Heritage Western Cape Provincial body of South Africa (permit # 2010/07/003). Precaution was made to minimize contamination of the skeleton prior to excavation. Archeological examination of the burial site and osteological examination of the skeleton were performed at the University of Cape Town. Skeletal samples for DNA analysis were transported under the South African Heritage Resources Agency export permit (80/11/11/002/52) between A.B.S. and V.M.H. To minimize contamination with present-day human DNA the extraction and library preparation were performed in a clean-room facility within the ancient DNA laboratory at the Max Planck Institute for Evolutionary Anthropology as per published procedures (Pääbo et al. 2004), followed by paired-end sequencing. A total of 30.4 mg powder from the internal root canal region of the tooth and 233.4 mg bone powder from the rib were used for DNA extraction as described (Rohland and Hofreiter 2007a, 2007b). Sequencing libraries were prepared as previously described (Meyer and Kircher 2010; Kircher et al. 2012), with the following modifications: 1) After indexing, eight amplification cycles were run for the library originated from the rib and 12 for the one originated from the tooth and 2) subsequent to the amplification, both libraries were purified using Qiagen MinElute PCR Purification Kit and eluted in 30 µl Elution Buffer. Mitochondrial sequence capture was performed individually or in pool as described (Maricic et al. 2010), including a library from the rib and one from tooth, as well as two negative controls derived at DNA extraction and library preparation, respectively. Additional amplification using the Phusion High-Fidelity PCR Kit by New England Biolabs (20 and 16 cycles for the rib and tooth, respectively) and quantification using the DNA 1000 Kit by Agilent Technologies were performed prior to sequencing.

Sequencing was performed in a pool of equimolar amounts of each library on one lane of a 75-cycle double indexed paired-end Illumina Genome Analyzer IIx run (sequencing chemistry kit v4, cluster generation kit v4, recipe version v7.4) (Kircher et al. 2012) and generating 1,021,385 sequencing reads for the tooth and 623,912 for the rib. Ibis was used for base-calling (Kircher et al. 2009) and raw sequences were further processed as reported (Dabney and Meyer 2012). Using MIA (Mapping Iterative Assembler available at https://github.com/udo-stenzel/mapping-iterative-assembler, last accessed December 2013) mitochondrial genomes were assembled separately for each library. In brief, sequencing reads were aligned to rCRS, position-specific nucleotide misincorporation typical for ancient DNA was considered and a consensus sequence built. Sequencing reads with the same start and end coordinate (unique molecules) were collapsed. The alignment process was repeated using the consensus sequence as reference genome, until previously and newly created consensus sequences did not differ any longer. At each position, the consensus was deduced by taking the base with the highest quality score.

Assembled genomes were analyzed for percentage of mtDNA contamination from present-day humans using a method available within the MIA program and using the previously described 32 diagnostic position panel. Aligned sequencing reads were counted as “clean” when carrying the diagnostic sample base and “contaminating” when matching the state of the contaminant. As deaminated cytosine, also known as uracil, will be recognized as thymine by DNA polymerases, adenine instead of thymine will be incorporated in the newly synthesized strand during the amplification process. Damage-Patterns (available at https://bioinf.eva.mpg.de/damage-patterns, last accessed September 18, 2014) was used to characterize the amount of C→T substitutions at 5′-end and G→A substitutions at 3′-end of sequences and substitution frequencies calculated for each position of sequencing reads as the proportion of sequencing reads carrying a T where the rCRS carries a C.

All other analyses were performed within the Laboratory for Human Comparative and Prostate Cancer Genomics at the Garvan Institute for Medical Research (previously at the J. Craig Venter Institute). Multiple sequence alignment of the 525 complete mtDNA was performed using MUSCLE v3.8.3 (Edgar 2004) 270 with default parameters. Phylogenetic analyses were performed after exclusion for known mutational hotspots (two poly-C runs at positions 303–315 and 16182–16194, an AC run at 515–525, and the mutational hotpot at 16519) resulting in the analysis of 16,531 bases for complete genomes and 15,447 bases for the coding region. Phylogenetic trees were reconstructed using FastTree v2.1.7 (Price et al. 2010) with default parameters and rooted to rCRS (haplogroup H2a2a1) and visualized with FigTree v1.4.0 (http://tree.bio.ed.ac.uk/software/figtree/, last accessed September 18, 2014).

## Supplementary Material

Supplementary figures S1–S5 are available at *Genome Biology and Evolution* online (http://www.gbe.oxfordjournals.org/).

Supplementary Data
