# Supplementary material for: First Ancient Mitochondrial Human Genome from a Prepastoralist Southern African
Source: Genome Biol Evol. 2014 Sep 10;6(10):2647–53. doi: 10.1093/gbe/evu202 (PMC4224329; doi:10.1093/gbe/evu202)
Supplement: Supplementary Data [file supp_6_10_2647__index.html]

First Ancient Mitochondrial Human Genome from a Prepastoralist Southern African — Supplementary Data 

# First Ancient Mitochondrial Human Genome from a Prepastoralist Southern African

## Supplementary Data

files

**Files in this Data Supplement:**

- Supplementary Data - pdf file
