# Supplementary material for: First Ancient Mitochondrial Human Genome from a Prepastoralist Southern African
Source: Genome Biol Evol. 2014 Sep 10;6(10):2647–53. doi: 10.1093/gbe/evu202 (PMC4224329; doi:10.1093/gbe/evu202)
Supplement: Supplementary Data [file supp_evu202_AMG-GBE_GenomeReport-SuppFIGs.pdf]

Supplementary Material

First Ancient Mitochondrial Human Genome from a Pre-Pastoralist Southern African

Alan G. Morris, Anja Heinze, Eva K.F. Chan, Andrew B. Smith, and Vanessa M. Hayes

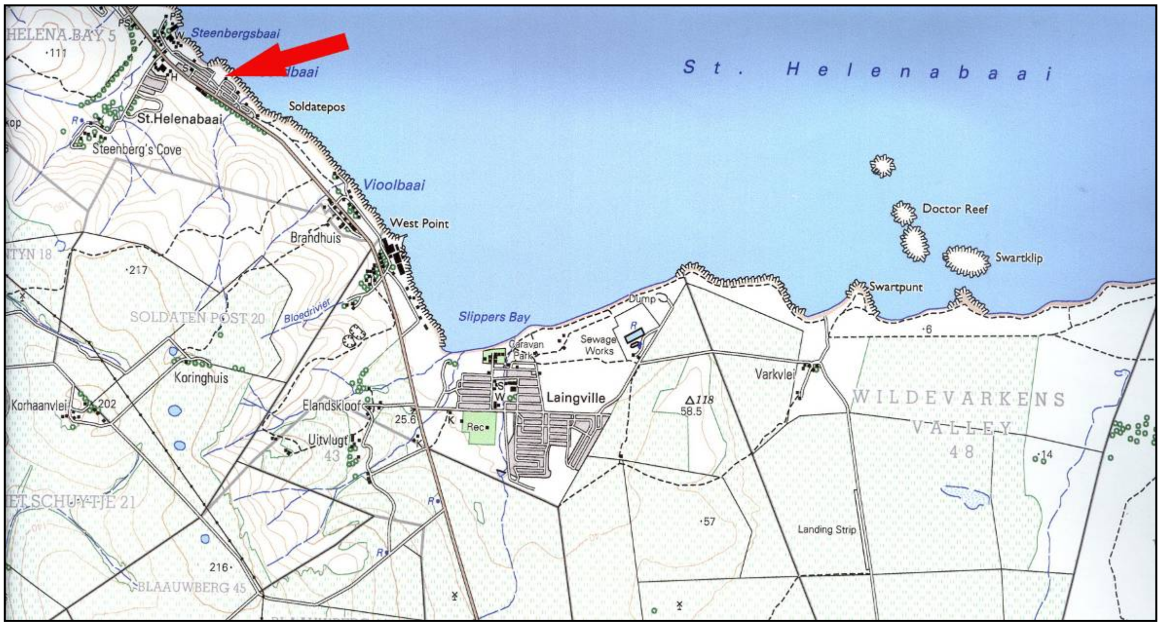

Figure S1. Map referenced location of the St Helena skeleton burial site. The skeleton was excavated from St Helena Bay (Figure S1) along the south-western coast of South Africa (32° 45’ 37S: 18° 01’ 47E on Erf 40, St Helena Bay Map Reference 3218 CA & CC).

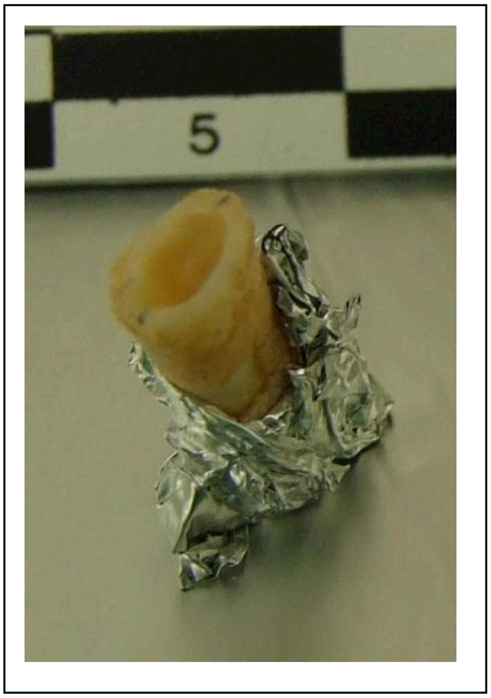

Figure S2. The tooth of the St. Helena Khoesan forager after generating powder from the inner canal region to minimize the impact of modern human contamination during extraction of ancient mtDNA.

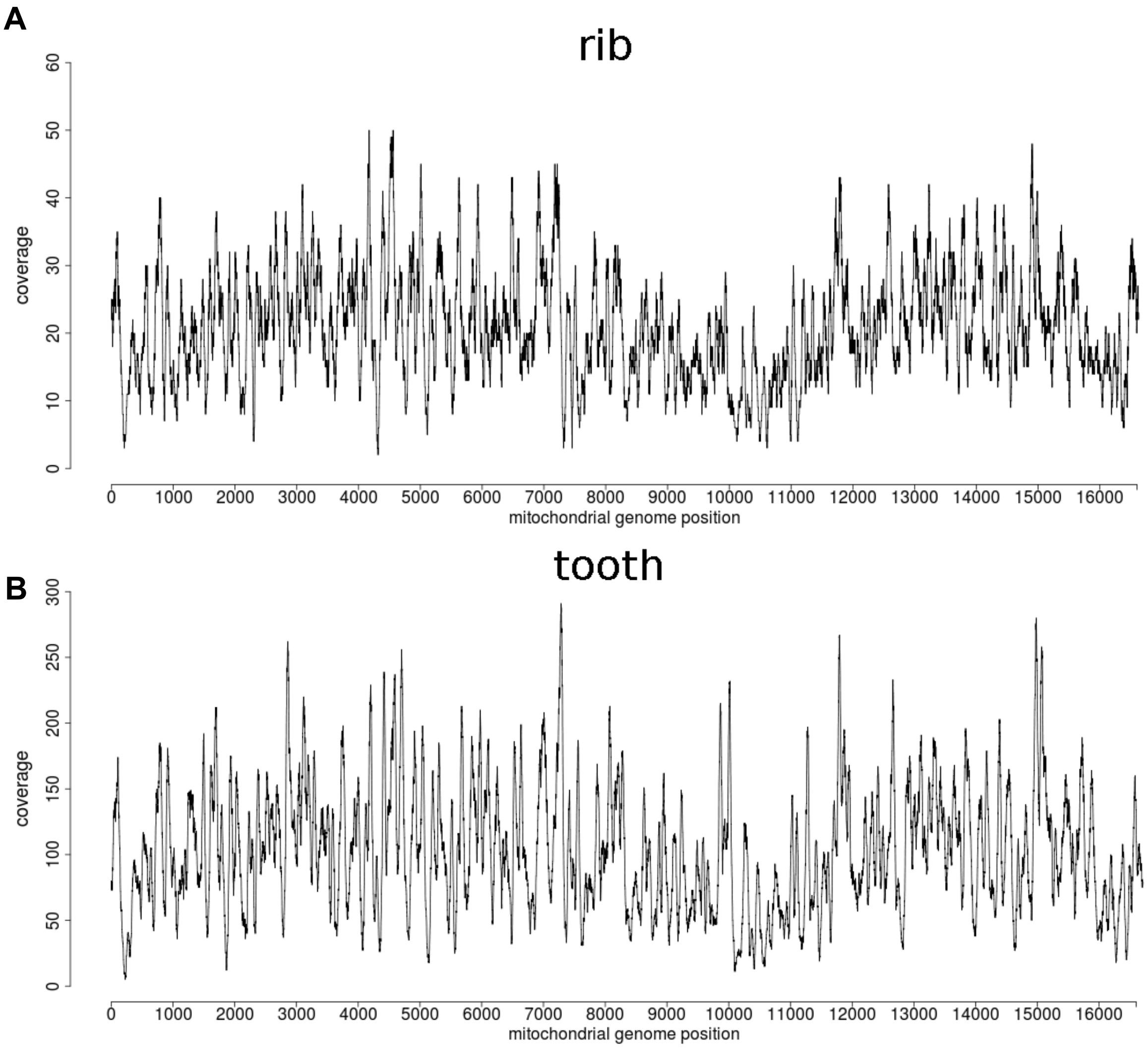

Figure S3. Sequencing coverage of the complete ancient mitochondrial genome originated from the **(A)** rib and **(B)** tooth of the St. Helena skeleton. After accounting for redundant copies of the same molecule, an average coverage of 20-fold for the rib and 103-fold for the tooth was achieved.

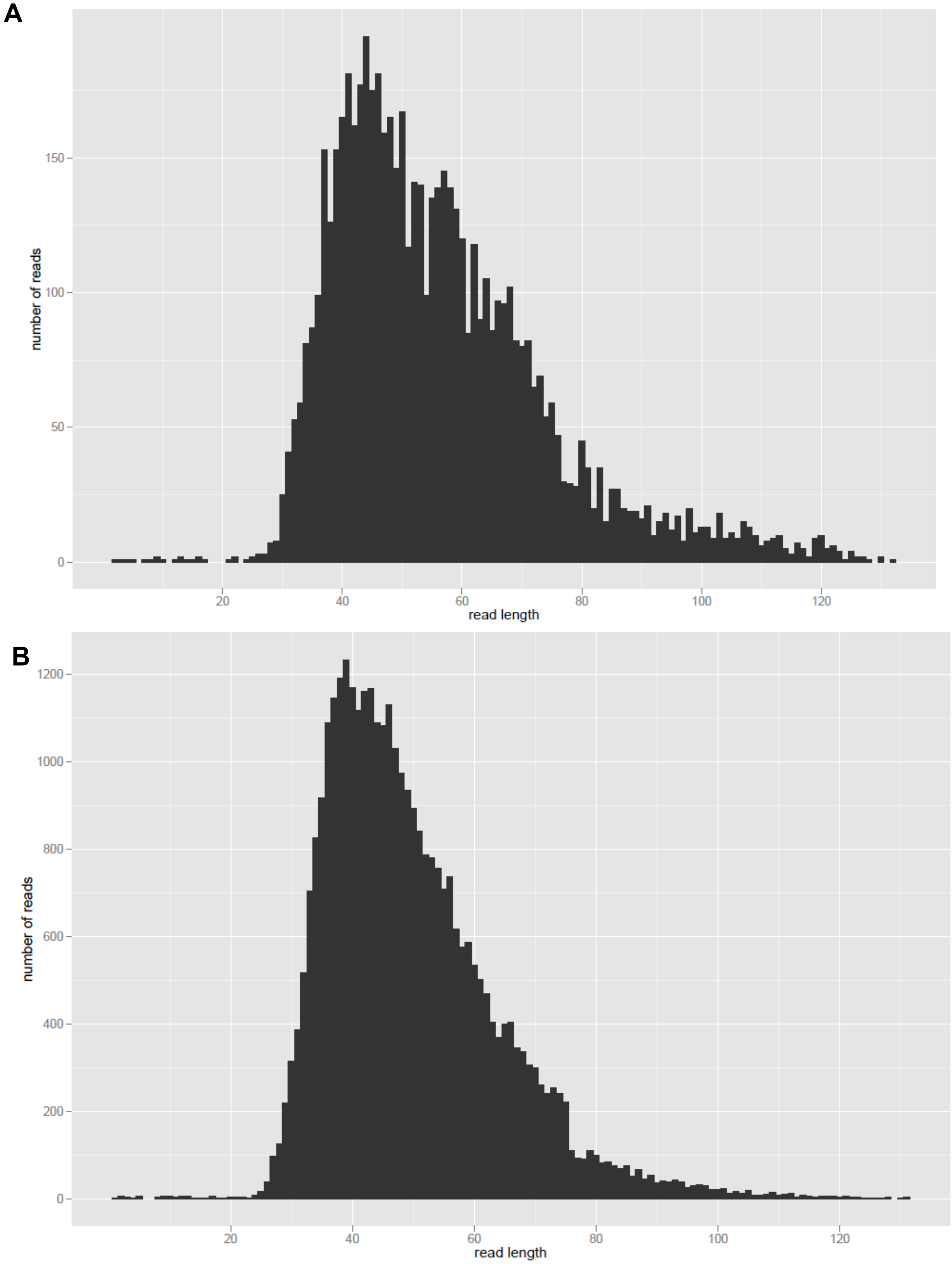

Figure S4. Fragment length distributions of sequencing libraries from St. Helena skeleton. The numbers of mitochondrial sequence reads per read length were counted for libraries originating from the (**A**) rib and (**B**) tooth after accounting for redundant copies of unique molecules arising from pre-sequencing amplifications. Mean fragment length is 50bp and 56bp for the tooth and the rib, respectively.

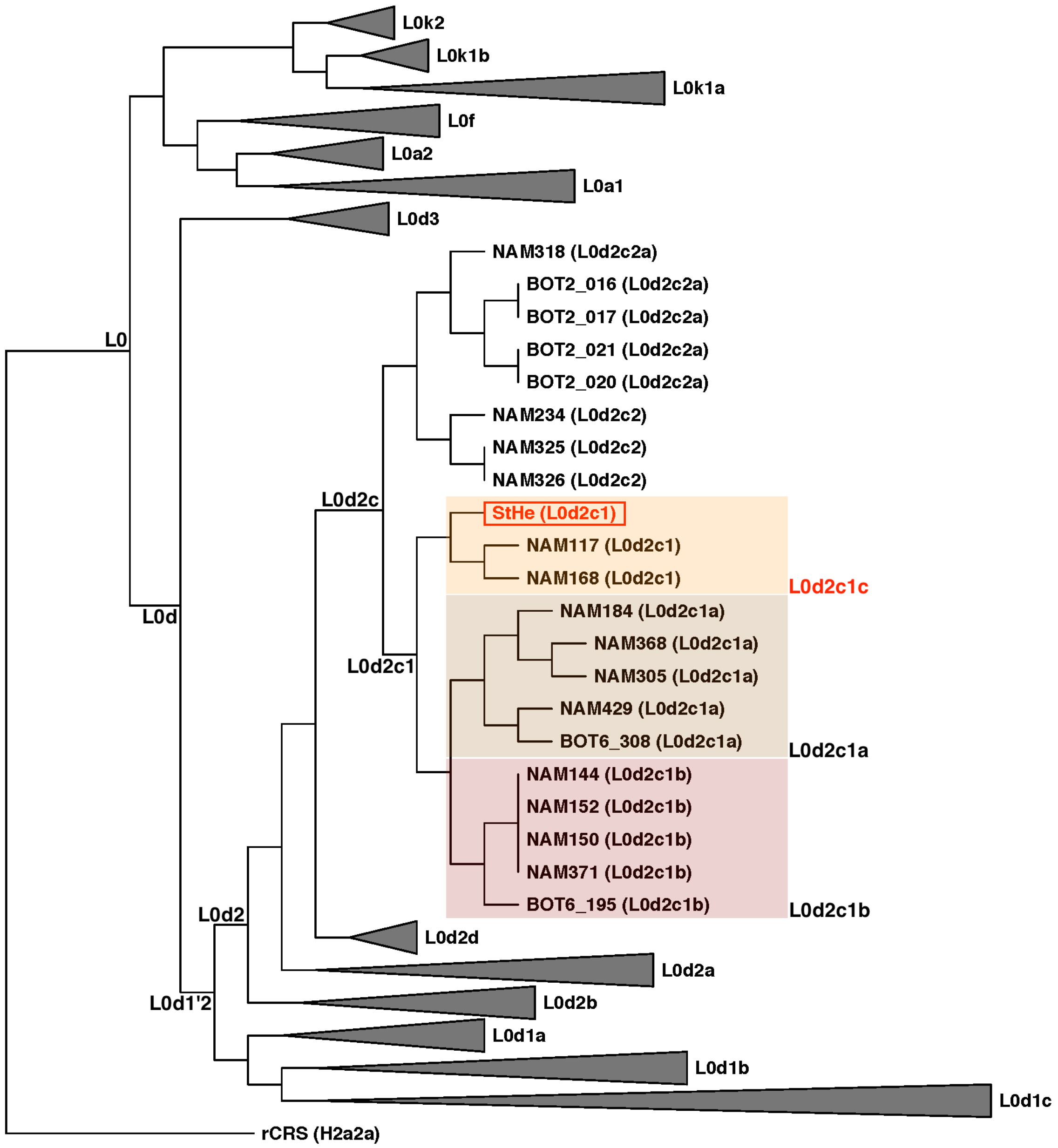

Figure S5. Phylogeny for the coding region of 526 mitochondrial genomes depicting the earliest diverged modern human maternal lineages, including the first ancient Khoesan mtDNA (StHe) within the L0d2c lineage. All non-L0d2c genomes have been collapsed with each triangle representing the relative diversity of the corresponding haplogroups and subclades.
